# Expression of Concern: The Uptake of Apoptotic Cells Drives *Coxiella burnetii* Replication and Macrophage Polarization: A Model for Q Fever Endocarditis

**DOI:** 10.1371/journal.ppat.1011029

**Published:** 2022-12-13

**Authors:** The PLOS Pathogens Editors

This article [1] has been identified as one of a series of submissions for which we have concerns about the reported research ethics approval information and the article’s adherence to PLOS research ethics policies.

PLOS will be investigating these concerns in accordance with COPE guidance and journal policies. Meanwhile, the *PLOS Pathogens* Editors issue this Expression of Concern.
